# Donor Perceptions and Preferences of Telemedicine and In-Person Visits for Living Kidney Donor Evaluation

**DOI:** 10.1016/j.ekir.2024.05.009

**Published:** 2024-05-15

**Authors:** Ellie Kim, Hannah C. Sung, Katya Kaplow, Victoria Bendersky, Carolyn Sidoti, Abimereki D. Muzaale, Jasmine Akhtar, Macey Levan, Suad Esayed, Amir Khan, Christina Mejia, Fawaz Al Ammary

**Affiliations:** 1 Department of Surgery, Johns Hopkins University School of Medicine, Baltimore, Maryland, USA; 2 Department of Surgery, NYU Grossman School of Medicine, New York, New York, USA; 3 Department of Medicine, University of California Irvine, California, USA; 4 Department of Medicine, Johns Hopkins University School of Medicine, Baltimore, Maryland, USA

**Keywords:** attitudes, delivery of health care, kidney transplantation, living donors, nephrectomy, telehealth

## Abstract

**Introduction:**

Living kidney donor evaluation is a lengthy and complex process requiring in-person visits. Access to transplant centers, travel costs, lost wages, and dependent care arrangements are barriers to willing donors initiating evaluation. Telemedicine can help streamline and epedite the evaluation process. We aimed to deeply understand donor experiences and preferences using hybrid telemedicine video/in-person visits to ease access to donor evaluation or counseling.

**Methods:**

We conducted in-depth, semistructured interviews with donors or donor candidates who completed their evaluation through telemedicine/in-person, or in-person only visits at a tertiary transplant center between November 27, 2019 and March 1, 2021. Enrollment continued until data saturation was reached (interviews with 20 participants) when no new information emerged from additional interviews. Transcripts were analyzed using inductive thematic analysis.

**Results:**

Eight themes were identified as follows: (i) reducing financial and logistical burdens (minimizing travel time and travel-related expenses), (ii) enhancing flexibility with scheduling (less time off work and child or family caregiver arrangements), (iii) importance of a walkthrough and establishing shared understanding, (iv) supporting information with technology and visual aids, (v) key role of the coordinator, (vi) preferred visit by provider role (meeting donor surgeon in-person to create rapport and engaging primary care provider in donor evaluation/follow-up), (vii) comparing modality differences in human connection, and (viii) opportunity for family and support network engagement (allowing loved ones to be involved in telemedicine visits irrespective of geographic locations and pandemic restrictions).

**Conclusion:**

Telemedicine/in-person hybrid model can make donor evaluation more accessible and convenient. Our findings help inform about determinants that influence the adoption of telemedicine to initiate donor evaluation to motivate willing donors. In addition, our results call for policy and legislation that support telemedicine services for living donor kidney transplantation across states.

## Graphical abstract

**Figure:**
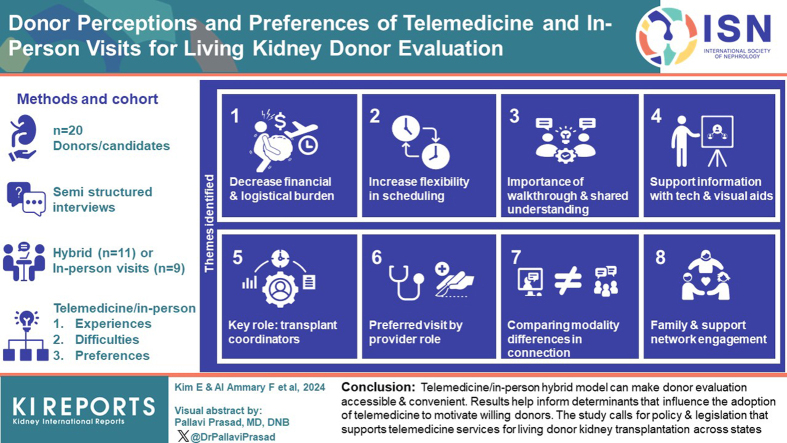

Living kidney donor evaluation is a lengthy and complex multiphase process for willing donors.^1^, ^2^, ^3^, ^4^ It can be difficult to engage willing donors to initiate their evaluation and counseling with in-person outpatient visits due to challenges with access to a transplant center, donor travel costs, lost wages, and dependent care arrangements.^5^, ^6^, ^7^, ^8^ As such, the initial in-person visits may exacerbate geographic, financial, or logistical barriers for willing donors. A study found that African American willing donors are 38% less likely to progress from medical screening to initiate the donor evaluation visit.^9^ Willing donor challenges might have contributed to the declining number of living kidney donors in the USA.^10^, ^11^, ^12^ Adopting an effective hybrid telemedicine/in-person care model for donor evaluation can streamline and expedite the process, and motivate willing donors to initiate the donor evaluation visit.^10^, ^11^, ^12^

The practice of telemedicine via real-time video visits during the COVID-19 pandemic demonstrated the potential of telemedicine to enhance access to transplant centers.^13^, ^14^, ^15^ In a national survey of multidisciplinary providers from 128 unique USA transplant centers, providers across roles agreed that telemedicine was efficient for transplant centers and convenient and accessible for donors, including those who have limited access (e.g., distance) to a transplant center, have limited financial or caregiving support, or reside out-of-state.^16^ In primary care studies, telemedicine video visits have achieved high levels of patient satisfaction and similar outcomes compared to in-person visits.^17^, ^18^, ^19^ Although telemedicine health care delivery may enhance current donor evaluation practice, it introduces new challenges. An in-depth understanding of donor experiences and preferences using telemedicine and in-person visits is critical to advance care for donors.

To deeply understand donor perceptions and preferences using hybrid telemedicine/in-person visits in the donor evaluation process, we conducted a qualitative research study. We aimed to describe themes focusing on the added value that telemedicine may provide over in-person only visits and areas for improving telemedicine to help enhance access to transplant centers and better adopt a hybrid telemedicine/in-person care model for donor evaluation and counseling.

## Methods

### Study Design

We conducted an inductive thematic qualitative study.^20^ We defined telemedicine as health care delivery of clinical services over distance using synchronous video visits. A hybrid model combining telemedicine and in-person visits for living kidney donor evaluation, where the willing donor initiates evaluation and counseling with a transplant nephrologist, surgeon, and coordinator via video visits (1-to-1 visit with each provider), then comes for a concise in-person visit to complete a physical examination and outstanding diagnostic testing; for example, CT scan and isotope measured glomerular filtration rate. We followed the Consolidated Criteria for Reporting Qualitative Research in reporting the methods and results (Supplementary Material).^21^

### Participant Selection and Setting

**Figure 1:**
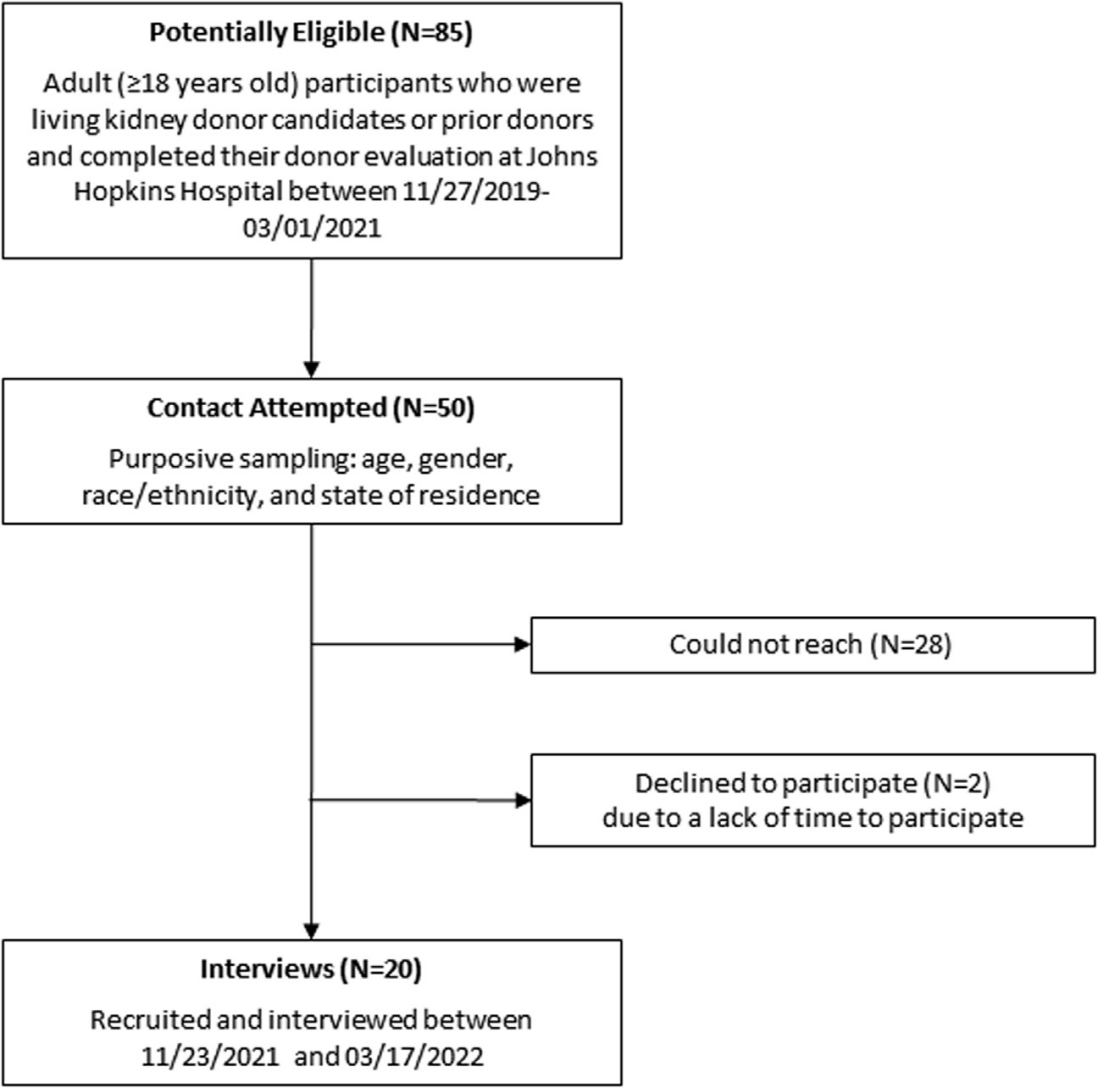
Flow diagram of study participants.

We included adult (aged ≥18 years) participants who were living kidney donor candidates or previous donors who were deemed donor eligible and completed donor evaluation at Johns Hopkins Hospital between November 27, 2019 and March 1, 2021 (Figure 1). Participants had hybrid telemedicine/in-person visits (*n* = 11) or in-person only (*n* = 9) visits modality. Two participants who initiated their donor evaluation in the hybrid model did not progress to in-person visits because they were deemed ineligible to be candidates after their video visits. We used purposive sampling based on age, gender (self-identified), race/ethnicity, and state of residence, to ensure diversity of perspectives on the study topic of telemedicine and in-person visits for living donor evaluation. The out-of-state purposeful sampling was to understand the impact of telemedicine licensing restrictions on out-of-state individuals. Two authors (FAA and JA) recruited participants by phone or email. We reminded participants during recruitment and consent that participation in the study was completely voluntary and would not affect their clinical care. Participants each received a $50 Amazon gift card for study participation. Verbal informed consent was obtained in accordance with the Johns Hopkins Institutional Review Board (IRB #00283653).

### Data Collection

We conducted in-depth, semistructured interviews. Our interview guide comprised questions related to the following: (i) experiences with telemedicine or in-person donor evaluation visits, (ii) difficulties to complete donor evaluation, and (iii) preferences for potential future telemedicine visits and suggestions to improve telemedicine in donor evaluation. The interview guide was developed based on a literature review and discussions with transplant experts and revised after pilot testing (Supplementary Appendix). The research team had trained in qualitative research methods. Training included measures to minimize the potential influence of interviewers on the interview dynamic. Interviewers, including clinical providers, introduced themselves as researchers and clarified that they were adopting a nonclinical role for the interview. Each participant had a single interview, 1-on-1, with a researcher. Two authors (FAA and CS) conducted the interviews between November 23, 2021 and March 17, 2022.

Study enrollment continued until data saturation was reached with each modality and no new information emerged from additional interviews. The interviews were audio- and video-recorded and lasted approximately 45 to 60 minutes. No further comments or feedback were added after participant interview completion. Interviewers created memos, and recordings were reviewed to verify the integrity and quality of the data. The audio recordings were professionally transcribed and deidentified for data analysis.

### Data Analysis

The authors (FAA, EK, HCS, KK, and VB) began with a data review, which informed the development of the codebook. We iteratively developed codes identifying different aspects of the donor evaluation process (e.g., expenses, scheduling, and use of technology). The authors (FAA, EK, KK, and VB) independently coded the data with 2 coders per transcript and resolved disagreements via consensus. We identified themes within and across codes until thematic saturation. Themes were then finalized through group discussions to reach a consensus. NVivo 13 (QSR International, Burlington, MA, 2020), a computer-assisted qualitative data analysis software, was used to facilitate data management and analysis.

We used several techniques to enhance trustworthiness.^21^^,^^22^ For dependability and confirmability of data analysis; we (i) annotated each interview transcript, (ii) created analytic memos, (iii) discussed data with the research team, and (iv) developed meeting notes and mind maps. For the credibility and transferability of our findings, we collected feedback from clinicians specializing in living donor kidney transplantation outside of the study team and contextualized our main findings within previous research on living kidney donation and the challenges of using telemedicine faced by US transplant centers.^16^^,^^23^

## Results

We interviewed a total of 20 donors and donor candidates because data saturation was reached and no new information emerged from additional interviews. Participants completed their evaluation between November 27, 2019 and March 1, 2021. Participants mean age was 44 (SD: ± 9; minimum = 30 and maximum = 60) years, with only 3 participants aged 55 to 60 years. Most participants were White (55%) or Black (35%), and half were out-of-state residents (including 3 from Virginia; 2 from New Jersey; and 1 each from California, Montana, New York, Pennsylvania, and Puerto Rico) (Table 1). In-state residents experienced the majority of telemedicine/in-person visits, whereas out-of-state residents were more likely to come for in-person only visits. Donor nephrectomy was accomplished in 5 participants who completed telemedicine/in-person visits and 5 participants who completed in-person only visits. The median time (interquartile range) from the date of evaluation to donor nephrectomy was 8 (5–9) months and 8 months (7–12) for donors who had telemedicine/in-person visits and in-person only visits, respectively. Themes that emerged from participant interviews are described in the next sections; the representative quotes to support each theme are provided in Table 2.

**Table 1:** Study participants’ characteristics

Participants	*n* = 20
Age, mean (SD), yr	44 (9)
Female, *n* (%)	15 (75)
Race/ethnicity, *n* (%)	
White	11 (55)
Black/African American	7 (35)
Hispanic	1 (5)
Other^a^	1 (5)
Telemedicine/in-person visits^b^	11 (55)
In-person only visits	9 (45)
Education, *n* (%)	
High School graduate or equivalent	3 (15)
Some college	6 (30)
Bachelor's	5 (25)
Postgraduate	6 (30)
Marital status, *n* (%)	
Married	14 (70)
Divorced/separated	2 (10)
Single	4 (20)
Private insurance, *n* (%)	16 (80)
Intended recipient of kidney donation, *n* (%)	
Biologically related	10 (50)
Biologically unrelated	9 (45)
Nondirected	1 (5)
Donated, *n* (%)	10 (50)
Out-of-state residence, *n* (%)	10 (50)

a Other includes 1 donor self-identified as Arab.

b Nine participants had combined telemedicine and in-person visits. Two participants had only telemedicine visits and deemed not candidates to progress to in-person visits.

**Table 2:** Themes and illustrative quotations

Illustrative quotations, by themes (participant)
Reducing financial and logistical burdens
"Well, (1), I could work and just take an hour to do something like this; (2), I wouldn't have to drive anywhere, so that would save on gas, and you wouldn't have the stress of— I think we left at like 5 in the morning to get there. It would just remove a lot of the things like transportation, just that kind of anxiety of just even getting there. Doing something like this you could be at home and still working, and it wouldn't cost anybody anything." (205)
"I preferred it, just like I’m preferring this, so as not to have to come in and pay for parking and all the commute time, etc., when it’s the same information as being exchanged." (219)
Enhancing Flexibility with scheduling
"Yeah, that eliminates you looking for somebody to watch your child." (204)
"I think it would be completely different, and I think the punctuality would be a whole lot better, because the physicians are—they're completely aware, and sometimes they may still be with a patient a little longer, but someone isn't sitting in an office and it's quiet in there and they're just sitting in there and they're bored. It's just completely different versus if you're at home or if you're at work and you're sitting at your computer waiting on the physician to sign-in for your appointment through Zoom. You can still be doing things. If you're at work, you can still be tidying-up some work over here while you're waiting versus being inside of the hospital and you're just sitting in a room waiting." (212)
"For me I think I only had to take 1 day off. I don't remember exactly. Maybe 2, but it was just when I had to go to the hospital that I took time off, but, again, I'm pretty lucky, because I can work remotely in my field of work, so that aspect was a little bit different for me probably than other donors." (217)
Importance of a walkthrough and establishing shared understanding
"Kind of telling people what it’s about, the purpose of the call. And even giving them—if they don't know, giving them a summary of what a transplant might be at the beginning, so they know what to expect through the call." (216)
"You know how like in health care you have like training videos, because during the donor process they send you a bunch of paperwork with information, and I think most people _______________ <inaudible 00:17:28> like reading, I think a video will, it's a good idea, because they send you a bunch of paperwork for you to read over, I think the social worker sends that, and I think a video, yeah, a video. I'm a visual learner." (218)
“I would say that I just generally have privacy concerns around using phones, cameras, and web cameras and stuff like that. I’m one of the people who’s, I would say, more conservative about that than others, but that might be typical. So, if it’s something like confidential, I would consider some sort of physical exam to be more confidential, then it’s not something I typically want to do on camera." (222)
Supporting information with technology and visual aids
"I personally am used to looking at the screen and seeing research or seeing diagrams or whatever as part of meetings that I do for work. So doing it on a telemedicine call, I do find helpful. But I'm also used to seeing that kind of stuff as part of the meetings we hold in our work because all our work right now is all remote." (207)
"I think that could be helpful. I think it would be helpful to ask first because some of my friends were looking at like, looking up on YouTube, how to watch videos of a kidney donor surgery happening. And I felt like I would be, too– that would be too stressful for me. But even just like a diagram picture of where incisions were made or what the process would look like I think just asking to offer that information if the patient is interested, that could be helpful." (209)
Key role of the coordinator
"I felt like I had been fully prepped by my care coordinator, and all of the materials that she had sent home that I had read through." (208)
"So, it was really just talking with my coordinator, and letting them know when I was available, and then picking a date. And then she bounced it off with her schedule, so she could have all the team available to meet with me. And then once we settled on a date and time, it was really—the gist of that, so it was pretty easy." (221)
Preferred visit by provider role
"I think just in the lead-up, maybe having a little bit of anxiety leading up to the procedure, having had met the surgeon in person rather than that having been virtual was just, I think just the—having a personal connection felt more sincere or felt more—I just had a little more confidence, I think, I’m guessing, than I would have if I hadn’t met him in person first." (213)
"I trust my primary care doctor, so I trust that she will do what she needs to do medically. And I think it’ll be great if she could send you the information, that way that you’ll have everything. The nephrologist or the surgeon, whoever the physician might be, will have all they need so we can have a more sufficient and productive telemedicine." (216)
"Well, we can't do X-rays and things like that through a video visit, so by being in person I was able to accomplish all those blood draws and things that have to be done in person all at the same time, so that was an advantage for sure. But I'm sure that like interviews with the social worker or the psychiatrist could have been done in a video chat if we'd been faced with that, but I wouldn't have minded at all." (203)
Comparing modality differences in human connection
“Yeah, I think probably I just have an inherent trust of I've been seen in person. They know me better. Maybe I would think of—I would get to meet the surgeon in person who instead of just meeting them the day before the surgery. I think that would help me to maybe get a better sense of their interaction, or their interpersonal—if I would feel like I was heard and listened to.” (209)
"Well, you get to meet the people. You get to see them. You can ask any questions you want while you’re on video stream. That’s the advantage you have. You actually get to see them and speak to them like you was in person." (204)
"Well, I think you didn’t have to wear a mask, so you could be face to face, and if you had any questions, maybe you could ask them at that time, and you feel like they’d put that time aside for you so they’re not in between patients coming in and out of lab rooms. You might feel like you have a little bit more time to ask any questions. I think that would be something positive." (220)
Opportunity for family and support network engagement
"Again, for a life-changing discussion. For a yearly checkup I don't know, but I think when you're dealing with such things as a transplantation—again, it was internal to my family, so it was a family affair. It was between my donating a kidney to my daughter, but my wife was ending up having 2 people in the family affected, so she wanted to be part of it, and, again, the opportunity to have significant others attend I think should be always offered." (210)
"I liked the fact also that my husband was able to kind of listen and then get in and ask questions, where normally he wouldn’t have been able to maybe get the time off of work if I had to go to the hospital." (215)

### Reducing Financial and Logistical Burdens

Participants commonly viewed telemedicine as more “convenient” than in-person visits, regardless of the modality that participants experienced. Many participants who had telemedicine/in-person visits described that telemedicine visits reduced travel hurdles compared with in-person visits. For example, participants mentioned fewer travel-related expenses related to telemedicine, such as not needing to pay for airplane tickets, gas for cars, and hospital parking. Further, a participant said, “I think more telemedicine would be awesome, very useful. Anything that helps folks access care more easily, so not too much to add.” Participants who had only in-person visits also envisioned that telemedicine would reduce travel costs as well as stress and anxiety about traveling to the center. In addition, they perceived telemedicine as “absolutely critical” and said they would have opted for telemedicine if it was allowed for their out-of-state residence. A participant stated, “I was living like 45 minutes from the Nevada state line, so I was like ‘I could drive to Nevada if it makes it better, or Oregon or any other state,’ so I had offered to do that too so I could do some of the evaluation with telemedicine, but it ended up that I just went to Maryland.”

### Enhancing Flexibility With Scheduling

Participants who had in-person/telemedicine visits perceived that scheduling was faster for telemedicine visits, and telemedicine visits took less time to complete compared with in-person visits. Participants across all visit modalities noted that telemedicine visits allowed for more flexibility when scheduling appointments, particularly for those who were caregivers of children or other family members. For instance, a participant who had an in-person visit shared that she had 3 children, and telemedicine would have been “much more convenient than getting childcare and organizing the family around doctors’ appointments.” Another participant postulated that stress from determining the logistics of having to find childcare and family care could prevent a donor candidate from completing their evaluation. Moreover, participants across all visit modalities said that telemedicine visits would make it easier for donor candidates to take less time off work. For instance, a participant who had both in-person and telemedicine visits explained: “So, if it was a 1-hour call, I was only taking an hour of leave. Whereas if it was in person, I would have– I would take at least 2 hours or 3 hours, because I'd have to drive down, go the appointment.”

### Importance of a Walkthrough and Establishing Shared Understanding

Participants across all visit modalities mentioned that they wanted more information to be given to them before their visit. A few participants said that they would have liked to have an introductory video or discussion with nonphysicians on everything they need to know for the visit. This included a walkthrough of the transplant center, the donation process, and testimonials from previous donors that can speak to the strengths of the transplant center and the rewards of organ donation. A participant who had an in-person visit believed that donors would feel more mentally prepared for the visit if they were told in advance that they would have to spend a full day at the hospital. Moreover, some participants who had telemedicine visits noted that they would have liked to be informed that they were going to be asked to show their body on camera before the visit. A few participants shared concerns about electronic records or video visits being hacked. Despite these concerns, most participants were able to find a private space at home or at work and did not express concerns about the security of virtual visits.

### Supporting Information With Technology and Visual Aids

Participants across all modalities discussed the overwhelming quantity of information they received during their visits. Some participants felt that there was a lot of information shared during the evaluation and that it was difficult to absorb it all at once. These participants noted that it was difficult for them to come up with questions to ask, and they only thought of questions after the fact. Some participants stated that telemedicine technology could improve information sharing. For instance, a participant described that technological features such as screen-sharing could be a benefit of having virtual visits, noting that sharing a screen may be more difficult in-person. A few participants suggested that using visual aids such as diagrams, pictures, and videos may also help with information sharing. A participant recalled that the nephrologist showed them a deck of slides describing research on donors and perceived that it provided context for the participant. Others noted that a video describing the technologies that would be used or where the surgical incision would be would have helped them formulate questions for the providers. A participant mentioned that a video of the procedure would be too stressful, but that general diagrams and pictures would be helpful.

### Key Role of the Coordinator

Participants across all modalities highlighted the importance of the coordinator role and explained that the coordinator’s role was to guide them through the process and facilitate their appointments. Participants also recalled that their nurse coordinator helped make the scheduling process easier for them. A few participants noted that meeting with the nurse coordinator role helped “set the tone” for the information shared later by the provider and helped them feel prepared for their visit. A participant felt that they “had a relationship (with the coordinator)” and when they got to meet the coordinator in-person, it “felt like a person (they) knew already.”

### Preferred Visit by Provider Role

Participants across all modalities had varying thoughts on which portions of the evaluation should be completed in-person versus via telemedicine visits. Some participants felt that the conversational portions of the evaluation could be completed via telemedicine, whereas portions that required physical examination should be performed in-person. Some reported that it was important for the provider, particularly the surgeon, to be able to touch the patient. Many participants stated that they needed to meet the surgeon in person to build a “connection” or “create a rapport.” Furthermore, some participants felt that engaging primary care providers in donor evaluation helps support the evaluation process and postdonation follow-up care. In addition, many participants preferred telemedicine for donor follow-up care; a participants said, “I went for an in-person follow-up, what was it, a week or 2 afterward and I’m glad I had an in person because I wanted someone to see my incision. . . but I haven’t had any consultations since then. I’ve just had lab work since then. So, if after that, anyone wanted to talk to me or if we wanted to have any follow-up assessments at 6 months or a year or anything like that, I would think a video visit would be completely called for and definitely the preferred way for me to do that.”

### Comparing Modality Differences in Human Connection

Participants across all modalities noted differences in communication and the feeling of personal connection when completing a visit via telemedicine versus in-person. Some found that a benefit of going in-person was being able to speak with others as they normally would. A participant said that doing things over video was “strange” and that they were used to the in-person format. Another participant stated that meeting their provider in-person “felt more sincere” and gave them “confidence.” Other participants felt no difference in communication between visit types. Some participants described the advantages of telemedicine for communicating with their providers. For example, a participant said that not having to wear masks during the pandemic made it easier to communicate with providers over video.

### Opportunity for Family and Support Network Engagement

Several participants, across modalities, recalled positive experiences with bringing a significant other or family member with them to either their in-person or telemedicine visit. They felt that having their significant others present was comforting and supportive, especially for life-changing decisions. A participant said that telemedicine allowed their partner to attend their visit regardless of pandemic-related restrictions. A participant who had an in-person visit mentioned that their significant others took time off work to attend the visit. However, another participant mentioned that telemedicine visits would allow significant others to attend because they would be able to take less time off work. A few participants explained that telemedicine would make it more convenient for significant others to attend evaluation visits, which would help participants process the information and come up with questions to ask.

## Discussion

In this qualitative study, donor and donor candidate participants shared their experiences and preferences regarding the capacity and role of telemedicine/in-person visits in donor evaluation. Participants perceived several benefits to telemedicine as being more accessible and convenient than in-person only visits. Participants noted the overwhelming amount of information they received during donor evaluation and suggested a role of telemedicine visits could be to communicate expectations related to donor evaluation, risks, and follow-up care. Participants indicated that the initial telemedicine visits could be used to alleviate concerns about the surgical procedure and donor outcomes. Further, participants indicated that telemedicine allowed them to bring their loved ones to evaluation-related visits irrespective of the geographic location or pandemic restrictions. Nonetheless, participants express the constraints of telemedicine regarding the physical examination and human connection, in addition to out-of-state licensing restrictions. Overall, our identified themes illuminate the value of telemedicine in reducing logistical challenges, facilitating counseling or knowledge-sharing with donors, and expanding opportunities for loved ones to be directly involved in the evaluation process, as well as highlighting areas for improvement.

This study's findings are consistent with previous perspectives of transplant providers across specialties that telemedicine services are accessible and convenient for donors.^16^ Our findings about the potential impact of telemedicine in reducing financial and logistical hurdles parallel the recommendations from the consensus conference of the American Society of Transplantation calling for strategies to reduce financial barriers to living kidney donation.^24^ Others reported that minimizing disincentives would support equity and justice in living kidney donation.^25^^,^^26^ Notably, our study shows that most participants from out-of-state had to come for only in-person visits, underlining the unsuitable state legislation restrictions for out-of-state licensed transplant providers, which further magnify disparities in access to living donor kidney transplantation.^27^ Enhancing access to transplant centers via telemedicine can help motivate willing donors to initiate their evaluation and ultimately complete the kidney donation and receive optimal follow-up care.^28^, ^29^, ^30^, ^31^

Our study highlights areas for clinical practice to better adopt telemedicine in donor evaluation and counseling. First, whereas the hybrid telemedicine/in-person model mitigates the limitation of telemedicine physical examinations, our study reflects the need for creating better approaches to advancing virtual physical examinations in ways that can maximize telemedicine visits. Our previous national survey study indicated that half of nephrologists and surgeons were not willing to accept a remote completion of a physical exam.^16^ Therefore, innovative approaches are needed to incorporate a meaningful limited physical examination into telemedicine, which may prevent it from becoming a bottleneck for individuals who do not have easy access to initiate the evaluation at a transplant center. Second, participants emphasize the importance of meeting with the donor surgeon in-person, for the surgeon to touch them, to establish rapport, and reduce surgery anxiety. It is imperative that the hybrid telemedicine/in-person model should be flexible to accommodate individual needs and preferences. Third, engaging primary care providers in sharing a donor candidate's history or physical health information can accelerate and optimize telemedicine visits for donor evaluation. Connecting primary care providers with the transplant center not only supports the evaluation process but also improves the long-term follow-up care of donors. Fourth, participants recognized coordinators as primary communicators and information sharers, though not specific to telemedicine. Thus, coordinators may be particularly important in navigating and setting expectations for telemedicine/in-person visits. Fifth, advancing telemedicine’s technological features to improve human connections and take full advantage of the ability to include multiple attendees.

Quality measurements that are applied to in-person visits should apply to telemedicine visits. It is essential that telemedicine program implementation does not lead to unintended consequences for underresourced communities.^32^ Providers should also ensure that there is no risk of donor coercion when using virtual evaluations. Access to a reliable internet connection and electronic devices need to be supported for persons with low incomes or in rural areas, given that telemedicine health care delivery helps improve their access to transplant centers.^26^^,^^27^^,^^32^, ^33^, ^34^

Our study results need to be interpreted within its limitations. First, despite our purposive sampling, our sample underrepresented individuals who identified as Hispanic or other race/ethnic categories apart from Black or White. Second, although we did not exclude non-English speakers from our study, we did not have non-English speaking participants. Non-English speakers may have experienced different challenges accessing and utilizing telemedicine, which need further research exploration. Third, our sample underrepresents older individuals aged >60 years who may have difficulty using technology, and future studies may need to address the needs of this population. Fourth, our study participants initiated their evaluation via telemedicine during the pandemic, which may have influenced our findings because they may have been more inclined to favor telemedicine over in-person visits. Although the pandemic was the context for accelerated telemedicine health care delivery under the public health emergency, our findings are valuable beyond the pandemic as telemedicine video visit options are becoming more available to patients. Further, participants’ attitudes toward telemedicine may also have been influenced by socioeconomic factors.

That said, our study had several strengths. This is the first study that has qualitatively examined the perceptions of donor or donor candidates who used telemedicine in donor evaluation. Whereas the concept of results generalizability applies to quantitative studies, the concept of results transferability is used for qualitative studies, which refers to the extent to which qualitative findings could be achieved in other similar situations. Though our findings have been generated at a single center with specific characteristics, donor and donor candidates’ views of the benefits and challenges of telemedicine are highly transferrable to other contexts and useful considerations for other transplant centers that are interested in advancing or starting their own telemedicine practices. In addition, our robust qualitative research methods, adhering to dependability, confirmability, credibility, and transferability through the use of multiple coders, analytic memos, and contextualizing consensus, ensured the trustworthiness of our findings.

In conclusion, our study provides an in-depth understating of donors or donor candidates' perceptions and viewpoints and contributes to the growing evidence of the added value of telemedicine in kidney transplantation.^35^ The reported viewpoints show that telemedicine/in-person visits can help make donor evaluation more accessible and convenient. Our findings help inform determinants that influence the adoption of a donor-centered, hybrid care model to initiate donor evaluation to motivate willing donors. In addition, our results call for policy and legislation to support telemedicine services for living donor kidney transplantation across states. Future studies are needed to assess the impact of telemedicine services on donor outcomes.

## Disclosure

All the authors declared no competing interests.

## Supplementary Material

Supplementary File (PDF) Consolidated Criteria for Reporting Qualitative Research Checklist. Interview Guide.

## Footnotes

Supplementary File (PDF)
Consolidated Criteria for Reporting Qualitative Research Checklist. Interview Guide.
