# Supplementary material for: Donor Perceptions and Preferences of Telemedicine and In-Person Visits for Living Kidney Donor Evaluation
Source: Kidney Int Rep. 2024 May 15;9(8):2453–61. doi: 10.1016/j.ekir.2024.05.009 (PMC11328557; doi:10.1016/j.ekir.2024.05.009)
Supplement: Supplementary File (PDF) — Consolidated Criteria for Reporting Qualitative Research Checklist. Interview Guide. [file mmc1.pdf]

Supplementary Material

COREQ (COnsolidated criteria for REporting Qualitative research) Checklist

A checklist of items that should be included in reports of qualitative research.

Topic	Item No.	Guide Questions/Description	Reported on Page No.
**Domain 1: Research team and reflexivity**			
Personal characteristics			
Interviewer/facilitator	1	Which author/s conducted the interview or focus group?	5
Credentials	2	What were the researcher's credentials? E.g. PhD, MD	1
Occupation	3	What was their occupation at the time of the study?	1
Gender	4	Was the researcher male or female?	1, 5
Experience and training	5	What experience or training did the researcher have?	1, 5
Relationship with participants			
Relationship established	6	Was a relationship established prior to study commencement?	5
Participant knowledge of the interviewer	7	What did the participants know about the researcher? e.g. personal goals, reasons for doing the research	5
Interviewer characteristics	8	What characteristics were reported about the inter viewer/facilitator? e.g. Bias, assumptions, reasons and interests in the research topic	5, 6
**Domain 2: Study design**			
Theoretical framework			
Methodological orientation and Theory	9	What methodological orientation was stated to underpin the study? e.g. grounded theory, discourse analysis, ethnography, phenomenology, content analysis	2, 5, 6
Participant selection			
Sampling	10	How were participants selected? e.g. purposive, convenience, consecutive, snowball	4
Method of approach	11	How were participants approached? e.g. face-to-face, telephone, mail, email	4
Sample size	12	How many participants were in the study?	4, 6, 23
Non-participation	13	How many people refused to participate or dropped out? Reasons?	4, 23
Setting			
Setting of data collection	14	Where was the data collected? e.g. home, clinic, workplace	5
Presence of non-participants	15	Was anyone else present besides the participants and researchers?	5
Description of sample	16	What are the important characteristics of the sample? e.g. demographic data, date	6, 19
Data collection			
Interview guide	17	Were questions, prompts, guides provided by the authors? Was it pilot tested?	5
Repeat interviews	18	Were repeat inter views carried out? If yes, how many?	5
Audio/visual recording	19	Did the research use audio or visual recording to collect the data?	5
Field notes	20	Were field notes made during and/or after the inter view or focus group?	5
Duration	21	What was the duration of the inter views or focus group?	5
Data saturation	22	Was data saturation discussed?	5
Transcripts returned	23	Were transcripts returned to participants for comment and/or	5

Topic	Item No.	Guide Questions/Description	Reported on Page No.
		correction?	
**Domain 3: analysis and findings**			
Data analysis			
Number of data coders	24	How many data coders coded the data?	5
Description of the coding tree	25	Did authors provide a description of the coding tree?	5
Derivation of themes	26	Were themes identified in advance or derived from the data?	5, 6
Software	27	What software, if applicable, was used to manage the data?	6
Participant checking	28	Did participants provide feedback on the findings?	5
Reporting			
Quotations presented	29	Were participant quotations presented to illustrate the themes/findings? Was each quotation identified? e.g. participant number	7-10, 20-21
Data and findings consistent	30	Was there consistency between the data presented and the findings?	6-11
Clarity of major themes	31	Were major themes clearly presented in the findings?	6-11
Clarity of minor themes	32	Is there a description of diverse cases or discussion of minor themes?	11-13

Developed from: Tong A, Sainsbury P, Craig J. Consolidated criteria for reporting qualitative research (COREQ): a 32-item checklist for interviews and focus groups. *International Journal for Quality in Health Care*. 2007. Volume 19, Number 6: pp. 349 – 357

Supplementary Appendix

The Interview Guide

<u>Participant Experiences with In-person or Video Visit</u>

This first set of questions will ask you about your experiences during the donor evaluation process.

1. Have you ever had a video visit for living kidney donor evaluation?
    a. [If "No"] Were you ever given the opportunity to have your initial evaluation via a video visit?
        i. [If "Yes"] Could you please tell me more about that?

2. Have you ever had an in-person visit for living kidney donor evaluation?

3. What was your overall experience like with the [insert modality] visit during the donor evaluation process?
    a. <u>Follow up [if unclear]:</u> Could you tell me more about that?

4. How did it feel to communicate with your provider during a(n) [insert modality] visit?
    a. [If they had a video visit] <u>Follow up:</u> how did it feel sharing personal or medical information with your provider over video?
    b. [If they had an in-person visit] <u>Follow up:</u> If you had ever a video visit, how did it feel sharing personal or medical information with your provider over video?

5. What difficulties did you encounter having to do the visit via [insert modality]?
    a. <u>Probe for in-person visit:</u> timing availability for the appointment, time spent to complete the evaluation, transportation (need someone to help), care giving issue (childcare or other family member), costs, logistics
    b. <u>Probe for video-visit:</u> timing availability for the appointment, time spent to complete the evaluation, finding a private space, logging in and using the video platform, checking into and out of multiple appointments, finding a secure internet connection, using electronic devices (I.e., Smartphone or computer)

6. How can we improve the donor evaluation experience for future living kidney donors who do a(n) [insert modality] visit?

7. [If they had a video visit] What would be improved if you had your evaluation in-person?
    a. <u>Follow up:</u> what difficulties do you think you would encounter if you had your evaluation in-person?
    b. <u>Follow up:</u> what advantages do you think you would have by doing this via video visit?

8. [If they had an in-person visit] What would be improved if you had your evaluation using a video visit?

    a. Follow up: what difficulties do you think you would encounter if you had your evaluation using a video visit?

9. If you had to choose between a video visit and an in-person visit, what would you choose?
    a. Follow up: What are some of your reasons you would choose [chosen option]?

[If they had a video visit] Participant Views on Physical Exam

As you may recall, you had a limited physical exam during your video visit. Now, I would like to ask you about the physical exam process. This physical exam may have included a review of your blood pressure and body weight and height readings, and an assessment your overall health appearance.

10. Did you have any concerns or issues completing part of your physical exam via a video visit? If so, could you please elaborate?
    a. How would you feel about completing your physical exam with a primary care provider or a local provider instead of with the transplant team?

Closing Question

11. Do you have any additional comments or feedback that you would like to share with us about using a(n) [insert modality] visit for the donor evaluation?

Demographic Questions

12. Thank you for participating in this interview. I'm going to end by asking you a few closed-ended questions about yourself.
    a. What gender do you identify with?
    b. What is your race?
        i. What is your ethnicity? Hispanic, non-Hispanic?
    c. What state did you live in when you completed the donor evaluation process?
